# FDG PET/CT to Predict Recurrence of Early Breast Invasive Ductal Carcinoma

**DOI:** 10.3390/diagnostics12030694

**Published:** 2022-03-12

**Authors:** Joon-Hyung Jo, Hyun Woo Chung, Young So, Young Bum Yoo, Kyoung Sik Park, Sang Eun Nam, Eun Jeong Lee, Woo Chul Noh

**Affiliations:** 1 Department of Nuclear Medicine, Konkuk University Medical Center, Seoul 05030, Korea; 20180122@kuh.ac.kr (J.-H.J.); youngso@kuh.ac.kr (Y.S.); 2 Research Institute of Biomedical Science, Konkuk University School of Medicine, Seoul 05029, Korea; 0117652771@kuh.ac.kr (Y.B.Y.); kspark@kuh.ac.kr (K.S.P.); 20210415@kuh.ac.kr (W.C.N.); 3 Department of Surgery, Konkuk University Medical Center, Seoul 05030, Korea; 20090055@kuh.ac.kr; 4 Department of Nuclear Medicine, Seoul Medical Center, Seoul 02053, Korea; cateunjeong@naver.com

**Keywords:** radiomics, FDG PET/CT, early breast invasive ductal carcinoma, recurrence, metabolic tumor volume

## Abstract

This study investigated the prognostic value of FDG PET/CT radiomic features for predicting recurrence in patients with early breast invasive ductal carcinoma (IDC). The medical records of consecutive patients who were newly diagnosed with primary breast IDC after curative surgery were reviewed. Patients who received any neoadjuvant treatment before surgery were not included. FDG PET/CT radiomic features, such as a maximum standardized uptake value (SUVmax), metabolic tumor volume (MTV), total lesion glycolysis (TLG), skewness, kurtosis, entropy, and uniformity, were measured for the primary breast tumor using LIFEx software to evaluate recurrence-free survival (RFS). A total of 124 patients with early breast IDC were evaluated. Eleven patients had a recurrence (8.9%). Univariate survival analysis identified large tumor size (>2 cm, *p* = 0.045), high Ki-67 expression (≥30%, *p* = 0.017), high AJCC prognostic stage (≥II, *p* = 0.044), high SUVmax (≥5.0, *p* = 0.002), high MTV (≥3.25 mL, *p* = 0.044), high TLG (≥10.5, *p* = 0.004), and high entropy (≥3.15, *p* = 0.003) as significant predictors of poor RFS. After multivariate survival analysis, only high MTV (*p* = 0.045) was an independent prognostic predictor. Evaluation of the MTV of the primary tumor by FDG PET/CT in patients with early breast IDC provides useful prognostic information regarding recurrence.

## 1. Introduction

Breast invasive ductal carcinoma (IDC) is the most common subtype of breast cancer worldwide. Although the incidence of breast cancer has increased over the past decade, mortality has declined progressively because of earlier detection and improved treatment [1]. As a result, accurate assessment of the risk of recurrence after the initial treatment is important to the quality and quantity of a patient’s normal life expectancy.

Radiomics is an intensely discussed topic in the medical imaging field. It is an approach for analyzing complex imaging patterns to extract quantitative and reproducible information that cannot be recognized by human vision [2]. Given that tumor tissues are a complex collection of cells with diverse molecular properties, radiomic features are thought to serve as a useful tool for evaluating intratumoral heterogeneity and identifying potentially important information for cancer characterization [3,4]. Local image features extraction (LIFEx) software measures the radiomic features of the entire tumor burden using images from various medical imaging modalities, and has been shown to provide convenient radiomics analysis [5].

Fluorine-18 fluorodeoxyglucose positron emission tomography/computed tomography (FDG PET/CT) is a metabolic imaging modality that is used for various indications of breast cancer such as staging, restaging, and evaluation of the treatment response [6,7]. The prognostic value of FDG PET/CT for assessing the metabolic activity of breast cancer has also been investigated [8]. However, many studies have assessed tumor metabolic activity and prognosticated patients with advanced stages with mixed subtypes of breast cancer [9,10].

We investigated whether the radiomic features obtained from pretreatment FDG PET/CT can provide prognostic information about recurrence in patients with early breast IDC.

## 2. Materials and Methods

### 2.1. Patients

This retrospective study was approved by our institutional review board, which waived patient informed consent (IRB file #: 2021-10-050). The medical records of consecutive patients who were newly diagnosed with primary breast IDC after curative surgery with axillary lymph node staging at our center from January 2013 to June 2017 were reviewed. FDG PET/CT was performed as part of the routine cancer staging procedures. To evaluate early breast IDC, patients who had a pathological stage from T1N0M0 to T3N0M0 (stage I–II) according to the 8th edition of the American Joint Committee on Cancer (AJCC) staging manual were included [11]. Patients who received any neoadjuvant treatment before surgery were not included.

All patients underwent subsequent clinical follow-up at our center after surgery. Adjuvant chemotherapy, radiotherapy, hormonal therapy, and targeted therapy were offered according to the patient’s medical status. For adjuvant chemotherapy, an anthracycline-based regimen, an anthracycline followed by taxane, or a taxane-based regimen was administered. Patients with hormone-sensitive (receptor-positive) breast IDC were treated with hormonal therapy. A trastuzumab-based regimen was given to patients with human epidermal growth factor receptor-2 (HER2) positive breast cancer. Follow-up evaluation included mammography, ultrasound, contrast-enhanced CT of the chest and abdomen, and bone scintigraphy. FDG PET/CT and breast magnetic resonance imaging (MRI) were added when clinically necessary.

### 2.2. FDG PET/CT Imaging

PET/CT images were acquired using a GEMINI TF 64 PET/CT scanner (Philips Medical Systems, Cleveland, OH, USA). FDG PET/CT was performed in accordance with the Society of Nuclear Medicine and Molecular Imaging and European Association of Nuclear Medicine procedure guidelines for tumor imaging [12]. Regular quality control/quality assurance procedure was done to maintain the accuracy and precision of image quantitation. All patients fasted for at least 6 h. Blood glucose concentration was checked before PET/CT imaging (<120 mg/dL for nondiabetic patients and <200 mg/dL for diabetic patients). PET/CT images were acquired 60 min after intravenous injection of FDG (3.7 MBq/kg of body weight) to patients in the resting state. The axes of both PET and CT systems were mechanically aligned. A CT without intravenous contrast was performed from the skull base to the mid-thigh for attenuation correction and anatomic localization. The standardized CT protocol was 120 kV, 50 mA, tube-rotation time 0.5 s per rotation, pitch 0.83, and section thickness 4 mm. Immediately after CT, PET images were acquired for 1.5 min per frame using a conventional three-dimensional protocol.

### 2.3. Analysis of FDG PET/CT Radiomic Features

Two nuclear medicine physicians who had more than 15 years of experience in PET/CT and were unaware of the clinicopathological results other than the presence of breast cancer assessed the FDG PET/CT images. Any discrepancies were resolved by consensus. All PET images were reviewed using the LIFEx package (version 7.0.0) to calculate multiple radiomic features [5]. The PET images in the DICOM format were imported into LIFEx. Volumetric regions of interest (VOIs) were placed over areas of the primary breast tumor semi-automatically. At first, a fixed threshold of standardized uptake value (SUV) 2.5 within the contouring margin was used to define an accurate tumor margin. If the tumor margin was not correctly defined, the SUV threshold was adjusted. After that, the PET radiomic features were calculated automatically by the program. Because at least 64 voxels of VOI were needed to calculate texture-based (higher-order) features using LIFEx and many of the early breast IDC showed mild tumor metabolic activity, only histogram (first-order) features such as maximum SUV (SUVmax), metabolic tumor volume (MTV), total lesion glycolysis (TLG), skewness, kurtosis, entropy, and uniformity were evaluated for this study.

### 2.4. Statistical Analysis

Data were analyzed to determine the test performance in the setting in which pretreatment FDG PET/CT radiomic features could be used for predicting the recurrence of primary operable breast IDC. Recurrence included both local recurrence and distant metastasis. The primary endpoint was recurrence-free survival (RFS), which was assessed from the date of breast cancer surgery to the date of the first cancer recurrence. Patients who did not have cancer recurrence at the date of their last clinical follow-up were censored. For survival analysis, the cutoff values for categorizing low and high features were determined using receiver-operating characteristic (ROC) curve analysis. Univariate analysis of prognostic factors was performed using the Kaplan–Meier method, and the significance of differences between survival curves was evaluated using the log-rank test. Multivariate survival analysis to identify independent prognostic factors was performed using a Cox proportional-hazards model that included significant univariate variables. The software PASW Statistics for Windows (version 17.0; SPSS Inc., Chicago, IL, USA) was used for the analyses; *p* < 0.05 was considered to indicate significance.

## 3. Results

### 3.1. Patient Characteristics

A total of 124 female patients with early breast IDC were evaluated for this study. The clinical and pathological characteristics of the study patients are summarized in Table 1. As 83 of the 124 patients had less than 64 voxels of VOI for the primary tumor on FDG PET/CT, texture-based (higher-order) radiomic features could not be calculated using LIFEx.

### 3.2. Survival Analysis

Eleven of the 124 (8.9%) patients had a recurrence. The median follow-up times were 93.6 months (range 52.2–105.6 months) and 28.3 months (range 12.4–60.3 months) for patients without and with recurrence, respectively. Ten of the 11 patients with recurrence experienced disease progression within 5 years. For survival analysis, the cutoff values for categorization as lower and higher age, tumor size, Ki-67 expression, SUVmax, MTV, TLG, skewness, kurtosis, entropy, and uniformity were set at 59, 2.0 cm, 30%, 5.0, 3.25 mL, 10.5, 0.64, 2.35, 3.15, and 0.1, respectively. Each cutoff value was selected based on ROC curve analysis of our data.

**Figure 1:**
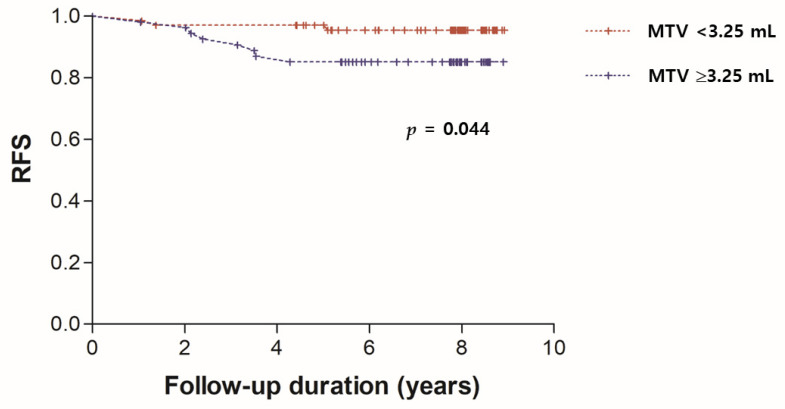
Kaplan–Meier analysis of recurrence-free survival (RFS) according to primary tumor metabolic tumor volume (MTV) value.

**Figure 2:**
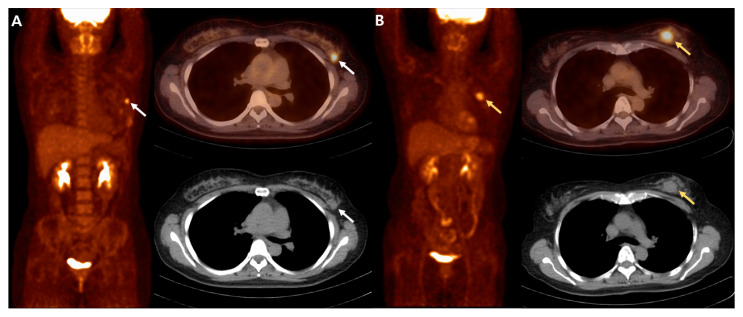
FDG PET/CT images in patients with early breast invasive ductal carcinoma. (**A**) A 50-year-old woman with AJCC anatomic stage T2N0M0 (IIA), AJCC prognostic stage IIA, primary tumor size 2.8 cm, SUVmax 7.5 and MTV 1.6 mL (white arrows). No recurrence was found until the last clinical follow-up of 94.6 months. (**B**) A 59-year-old woman with AJCC anatomic stage T2N0M0 (IIA), AJCC prognostic stage IIA, primary tumor size 2.5 cm, SUVmax 6.2 and MTV 4.4 mL (yellow arrows). Lung metastasis was found after 37.2 months of follow-up.

Univariate and multivariate analyses of RFS are demonstrated in Table 2. Univariate survival analysis identified large tumor size (>2 cm, *p* = 0.045), high Ki-67 expression (≥30%, *p* = 0.017), high AJCC prognostic stage (≥II, *p* = 0.044), high SUVmax (≥5.0, *p* = 0.002), high MTV (≥3.25 mL, *p* = 0.044), high TLG (≥10.5, *p* = 0.004), and high entropy (≥3.15, *p* = 0.003) as significant predictors of poor RFS. Therefore, multivariate survival analysis included tumor size, AJCC prognostic stage, Ki-67 expression, SUVmax, MTV, TLG, and entropy as variables. After multivariate survival analysis, only high MTV (*p* = 0.045) was an independent prognostic predictor of recurrence. The Kaplan–Meier survival curve for RFS of patients according to MTV is shown in Figure 1. Representative examples from patients with early breast IDC with low and high MTV are shown in Figure 2.

## 4. Discussion

Although anatomical TNM staging still provides important information for predicting an outcome for patients with breast IDC, the clinician must consider multiple factors such as tumor proliferation, histological grade, receptor phenotype, and genomic test results when determining treatment type and intensity. Selecting patients with a worse prognosis before treatment may influence treatment decisions and improve survival. In this study, the MTV of the primary tumor calculated using pretreatment FDG PET/CT was an independent prognostic predictor for recurrence in patients with early breast IDC who received the current standard therapy.

Imaging analysis of cancer is considered to have advantages for assessing the entire tumor compared with biopsies, which represent only a single anatomic site within the tumor [13]. Recent advances in medical imaging technology have led to rapid progress in image analysis. Traditionally, medical images have been used to evaluate qualitative features, such as tumor density, pattern of contrast enhancement, and regularity of tumor margins, called semantic features. By comparison, radiomics is a digital tool for decoding the information in medical images into quantitative features that are associated with tumor size, shape, intensity, and texture [14]. These features are thought to reflect the genetic and molecular mechanisms of tumors comprehensively and noninvasively [15].

Various software packages have been introduced for radiomic feature calculation. However, without standardization of the packages, the reliability of the features is of concern [16]. To address this concern, the imaging biomarker standardization initiative (IBSI) was developed as an international collaboration to help standardize radiomic features, with recommendations for standardized feature calculation, definition, and nomenclature [17]. LIFEx is an easily available freeware for making automatic calculations of radiomic features in medical images such as PET, CT, and MRI, without requiring specific programming expertise. LIFEx follows the IBSI-compliant calculation settings for standardization. Recent studies have used LIFEx to evaluate radiomics models for characterizing various cancers [18,19,20,21,22].

FDG PET/CT is a promising metabolic imaging modality that can provide more detailed complementary prognostic information compared with conventional anatomic imaging methods and clinicopathological factors. In the present study, the MTV of the primary tumor observed in pretreatment FDG PET/CT in patients with early breast IDC was the only independent prognostic factor for RFS. The other significant factors in the univariate survival analysis, such as tumor size, Ki-67 expression, AJCC prognostic stage, SUVmax, TLG, and entropy were not independent prognostic factors after the multivariate analysis. This result may reflect that the current standard therapy has been developed to improve patients’ outcomes with regard to previously validated prognostic factors that are not related to FDG PET/CT parameters. Therefore, this might emphasize the value of MTV in determining treatment as a prognostic predictor of recurrence in patients with early breast IDC. In patients with early breast IDC with a high MTV, neoadjuvant therapy or prolonged adjuvant therapy may be helpful for suppressing cancer recurrence after curative surgery.

Previous reports also suggested the MTV in pretreatment FDG PET/CT as a prognostic predictor of recurrence for breast cancer [23,24,25]. However, many of the patients in those studies had an advanced breast cancer. Furthermore, in a meta-analysis in patients with heterogeneous stages and subtypes of breast cancer, high MTV was not significantly correlated with recurrence [26]. Kitajima et al. reported that primary tumor SUVmax, MTV and TLG were significantly associated with recurrence in univariate survival analysis, but none were an independent predictor in multivariate analysis in patients with mostly early breast IDC [27]. That study had a fewer number of patients and shorter clinical follow-up than our study. Therefore, our study may demonstrate the prognostic value of the primary tumor MTV in patients with early breast IDC more significantly than other studies.

The SUVmax of the primary tumor was not an independent prognostic indicator of recurrence in this study. Other reports have suggested that tumor aggressiveness and survival are associated with SUVmax in patients with various cancers [28,29,30]. However, because SUVmax has only a single maximum uptake value in a region of interest, it has limited value for indicating the precise metabolic burden of the entire tumor. Thus, other recent reports have recommended that volumetric parameters obtained using FDG PET/CT, such as the MTV and TLG, have greater significant prognostic value than SUVmax alone, in patients with various cancers [31,32]. Consistent with these reports, our results suggest that the MTV of the primary tumor calculated by pretreatment FDG PET/CT may be a more accurate prognostic predictor of recurrence than SUVmax in patients with early breast IDC after curative surgery.

Our study has some limitations. First and most notably, it was a retrospective review with a limited number of patients and follow-up period; only 11 patients (8.9%) had recurrent breast IDC. A greater number of patients and longer follow-up would further validate the findings. However, because most of the recurrence happened within 5 years and most of the patients who survived without recurrence were followed for more than 5 years, it is unlikely that the conclusions of the present study would change significantly with a longer follow-up. Second, only histogram (first-order) radiomic features were evaluated in this study. Texture-based (higher-order) radiomic features showed significant associations with recurrence of breast cancer in several previous studies [33,34,35]. Higher-order features were suggested as a significant prognostic predictor for RFS more than first-order features in the studies. In this study, many of the patients with mild FDG uptake of the primary tumor on PET/CT showed less than 64 voxels in the VOI in the primary tumor. Higher-order radiomic features could not be measured because the LIFEx software requires a minimum of 64 voxels in VOI in the primary tumor. This was expected, because this study was designed to assess patients with early breast IDC who had a small tumor size and low stage. More advanced and sensitive software for radiomic feature calculation is essential for applying radiomics in a wider spectrum of cancers. Third, we did not evaluate whether FDG PET/CT parameter, such as the MTV, changes the treatment decision. Further prospective large-scale studies are needed to establish guidelines for any additional standard therapy.

## 5. Conclusions

The MTV of the primary tumor measured by pretreatment FDG PET/CT was an independent prognostic factor for recurrence in patients with early breast IDC. The MTV was a more reliable prognostic predictor than AJCC prognostic stage, Ki-67 expression, SUVmax, and entropy. Assessment of the MTV of the primary tumor by pretreatment FDG PET/CT provides useful information about the prognosis of recurrence in patients with early breast IDC.

## Figures and Tables

**Table 1:** Clinical and pathological characteristics of patients.

Characteristic	Value
Mean age (range)	60 ± 10 (38–86)
Type of surgery	
BCS ^a^ Simple mastectomy MRM ^b^	102 4 18
Mean primary tumor size (range)	1.9 ± 0.9 (0.3–4.7) cm
Histologic grade	
G1 G2 G3	16 58 50
AJCC ^c^ anatomic stage	
IA IB IIA IIB	70 0 41 13
AJCC prognostic stage	
IA IB IIA IIB IIIA IIIB IIIC	76 21 17 5 4 0 1
Receptor status	
Estrogen receptor (+/−)	103/21
Progesterone receptor (+/−)	73/51
HER2 ^d^ (+/−)	24/100
Mean Ki-67 expression (range)	29 ± 24 (1–95) %
Adjuvant chemotherapy (+/−)	95/29
Adjuvant radiotherapy (+/−)	101/23
Adjuvant hormonal therapy (+/−)	107/17
Mean SUVmax ^e^ (range)	5.2 ± 4.5 (0–19.2)
Mean MTV ^f^ (range)	3.7 ± 3.2 (0–22.3) ml
Mean TLG ^g^ (range)	13.3 ± 22.7 (0–198.5)
Mean skewness (range)	0.69 ± 0.39 (−0.40–1.60)
Mean kurtosis (range)	2.65 ± 1.03 (0–4.99)
Mean entropy (range)	2.57 ± 1.10 (0–4.80)
Mean uniformity (range)	0.18 ± 0.11 (0–0.48)

^a^ BCS = breast-conserving surgery, ^b^ MRM = modified radical mastectomy, ^c^ AJCC = American Joint Committee on Cancer, ^d^ HER2 = human epidermal growth factor receptor-2, ^e^ SUVmax = maximum standardized uptake value, ^f^ MTV = metabolic tumor volume, ^g^ TLG = total lesion glycolysis.

**Table 2:** Univariate and multivariate survival analyses for recurrence-free survival.

		Univariate Analysis			Multivariate Analysis		
Variables	No. of Patients	HR ^a^	95% CI ^b^	*p*	HR	95% CI	*p*
Age							
<60	64	1					
≥60	60	0.59	0.18−1.96	0.397			
Size							
≤2 cm	80	1					
>2 cm	44	3.27	1.03−12.26	0.045	0.55	0.09−3.44	0.519
Histologic grade							
I and II	74	1					
III	50	1.83	0.56−6.23	0.31			
N stage							
N0	101	1					
N1	23	0.42	0.12−2.34	0.395			
AJCC ^c^ anatomic stage							
I	70	1					
II	54	0.44	0.13−1.44	0.174			
AJCC prognostic stage							
I	97	1					
≥II	27	3.16	1.04−19.07	0.044	0.62	0.13−3.07	0.558
Estrogen receptor							
+	103	1					
−	21	1.92	0.45−11.02	0.326			
Progesterone receptor							
+	73	1					
−	51	1.77	0.54−6.02	0.337			
HER2 ^d^							
+	24	1					
−	100	Undefined		0.094			
Ki-67							
<30%	75	1					
≥30%	49	4.39	1.31−14.98	0.017	2.76	0.57−13.37	0.208
Adjuvant chemotherapy							
−	29	1					
+	95	Undefined		0.061			
Adjuvant radiotherapy							
−	23	1					
+	101	1.01	0.22−4.64	0.995			
Adjuvant hormonal therapy							
−	17	1					
+	107	0.38	0.04−1.56	0.142			
SUVmax ^e^							
<5.0	76	1					
≥5.0	48	7.71	2.03−23.52	0.002	6.66	0.54−82.84	0.14
MTV ^f^							
<3.25 mL	70	1					
≥3.25 mL	54	3.59	1.04−11.30	0.044	4.99	1.04−24.09	0.045
TLG ^g^							
<10.5	82	1					
≥10.5	42	5.54	1.77−22.12	0.004	2.65	0.37−19.03	0.332
Skewness							
<0.638	49	1					
≥0.638	75	1.30	0.39−4.40	0.663			
Kurtosis							
<2.35	36	1					
≥2.35	88	0.69	0.18−2.52	0.565			
Entropy							
<3.15	83	1					
≥3.15	41	5.78	1.89−23.99	0.003	1.41	0.163−12.11	0.757
Uniformity							
<0.1	36	1					
≥0.1	88	0.46	0.11−1.56	0.192			

^a^ HR = hazard ratio, ^b^ CI = confidence interval, ^c^ AJCC = American Joint Committee on Cancer, ^d^ HER2 = human epidermal growth factor receptor-2, ^e^ SUVmax = maximum standardized uptake value, ^f^ MTV = metabolic tumor volume, ^g^ TLG = total lesion glycolysis.

## Data Availability

The data presented in this study are available on request from the corresponding author. The data are not publicly available due to privacy limitations.

## References

[1] Sharma R. (2019). Breast cancer incidence, mortality and mortality-to-incidence ratio (MIR) are associated with human development, 1990–2016: Evidence from Global Burden of Disease Study 2016. Breast Cancer.

[2] Mayerhoefer M.E., Materka A., Langs G., Haggstrom I., Szczypinski P., Gibbs P., Cook G. (2020). Introduction to Radiomics. J. Nucl. Med..

[3] Vitale I., Shema E., Loi S., Galluzzi L. (2021). Intratumoral heterogeneity in cancer progression and response to immunotherapy. Nat. Med..

[4] Ha S., Choi H., Paeng J.C., Cheon G.J. (2019). Radiomics in Oncological PET/CT: A Methodological Overview. Nucl. Med. Mol. Imaging.

[5] Nioche C., Orlhac F., Boughdad S., Reuze S., Goya-Outi J., Robert C., Pellot-Barakat C., Soussan M., Frouin F., Buvat I. (2018). LIFEx: A Freeware for Radiomic Feature Calculation in Multimodality Imaging to Accelerate Advances in the Characterization of Tumor Heterogeneity. Cancer Res..

[6] Ulaner G.A. (2019). PET/CT for Patients with Breast Cancer: Where Is the Clinical Impact?. AJR Am. J. Roentgenol..

[7] Groheux D., Cochet A., Humbert O., Alberini J.L., Hindie E., Mankoff D. (2016). ^18^F-FDG PET/CT for Staging and Restaging of Breast Cancer. J. Nucl. Med..

[8] Paydary K., Seraj S.M., Zadeh M.Z., Emamzadehfard S., Shamchi S.P., Gholami S., Werner T.J., Alavi A. (2019). The Evolving Role of FDG-PET/CT in the Diagnosis, Staging, and Treatment of Breast Cancer. Mol. Imaging Biol..

[9] Groheux D., Giacchetti S., Delord M., de Roquancourt A., Merlet P., Hamy A.S., Espie M., Hindie E. (2015). Prognostic impact of ^18^F-FDG PET/CT staging and of pathological response to neoadjuvant chemotherapy in triple-negative breast cancer. Eur. J. Nucl. Med. Mol. Imaging.

[10] Cochet A., Dygai-Cochet I., Riedinger J.M., Humbert O., Berriolo-Riedinger A., Toubeau M., Guiu S., Coutant C., Coudert B., Fumoleau P. (2014). ^18^F-FDG PET/CT provides powerful prognostic stratification in the primary staging of large breast cancer when compared with conventional explorations. Eur. J. Nucl. Med. Mol. Imaging.

[11] Amin M.B., Greene F.L., Edge S.B., Compton C.C., Gershenwald J.E., Brookland R.K., Meyer L., Gress D.M., Byrd D.R., Winchester D.P. (2017). The Eighth Edition AJCC Cancer Staging Manual: Continuing to build a bridge from a population-based to a more “personalized” approach to cancer staging. CA Cancer J. Clin..

[12] Boellaard R., Delgado-Bolton R., Oyen W.J., Giammarile F., Tatsch K., Eschner W., Verzijlbergen F.J., Barrington S.F., Pike L.C., Weber W.A. (2015). FDG PET/CT: EANM procedure guidelines for tumour imaging: Version 2.0. Eur. J. Nucl. Med. Mol. Imaging.

[13] Gillies R.J., Kinahan P.E., Hricak H. (2016). Radiomics: Images Are More than Pictures, They Are Data. Radiology.

[14] Aerts H.J., Velazquez E.R., Leijenaar R.T., Parmar C., Grossmann P., Carvalho S., Bussink J., Monshouwer R., Haibe-Kains B., Rietveld D. (2014). Decoding tumour phenotype by noninvasive imaging using a quantitative radiomics approach. Nat. Commun..

[15] Bi W.L., Hosny A., Schabath M.B., Giger M.L., Birkbak N.J., Mehrtash A., Allison T., Arnaout O., Abbosh C., Dunn I.F. (2019). Artificial intelligence in cancer imaging: Clinical challenges and applications. CA Cancer J. Clin..

[16] Fornacon-Wood I., Mistry H., Ackermann C.J., Blackhall F., McPartlin A., Faivre-Finn C., Price G.J., O’Connor J.P.B. (2020). Reliability and prognostic value of radiomic features are highly dependent on choice of feature extraction platform. Eur. Radiol..

[17] Zwanenburg A., Vallieres M., Abdalah M.A., Aerts H.J.W.L., Andrearczyk V., Apte A., Ashrafinia S., Bakas S., Beukinga R.J., Boellaard R. (2020). The Image Biomarker Standardization Initiative: Standardized Quantitative Radiomics for High-Throughput Image-based Phenotyping. Radiology.

[18] Liu Z., Zhu G., Jiang X., Zhao Y., Zeng H., Jing J., Ma X. (2020). Survival Prediction in Gallbladder Cancer Using CT Based Machine Learning. Front. Oncol..

[19] Aksu A., Karahan Sen N.P., Acar E., Capa Kaya G. (2020). Evaluating Focal ^18^F-FDG Uptake in Thyroid Gland with Radiomics. Nucl. Med. Mol. Imaging.

[20] Sollini M., Kirienko M., Cavinato L., Ricci F., Biroli M., Ieva F., Calderoni L., Tabacchi E., Nanni C., Zinzani P.L. (2020). Methodological framework for radiomics applications in Hodgkin’s lymphoma. Eur. J. Hybrid Imaging.

[21] Zhang J., Zhao X., Zhao Y., Zhang J., Zhang Z., Wang J., Wang Y., Dai M., Han J. (2020). Value of pre-therapy ^18^F-FDG PET/CT radiomics in predicting EGFR mutation status in patients with non-small cell lung cancer. Eur. J. Nucl. Med. Mol. Imaging.

[22] Kocak B., Durmaz E.S., Ates E., Sel I., Turgut Gunes S., Kaya O.K., Zeynalova A., Kilickesmez O. (2020). Radiogenomics of lower-grade gliomas: Machine learning-based MRI texture analysis for predicting 1p/19q codeletion status. Eur. Radiol..

[23] Chang C.C., Chen C.J., Hsu W.L., Chang S.M., Huang Y.F., Tyan Y.C. (2019). Prognostic significance of metabolic parameters and textural features on ^18^F-FDG PET/CT in invasive ductal carcinoma of breast. Sci. Rep..

[24] Groheux D., Martineau A., Teixeira L., Espié M., de Cremoux P., Bertheau P., Merlet P., Lemarignier C. (2017). ^18^FDG-PET/CT for predicting the outcome in ER+/HER2- breast cancer patients: Comparison of clinicopathological parameters and PET image-derived indices including tumor texture analysis. Breast Cancer Res..

[25] Kim T.H., Yoon J.K., Kang D.K., Kang S.Y., Jung Y.S., Han S., Kim J.Y., Yim H., An Y.S. (2016). Value of volume-based metabolic parameters for predicting survival in breast cancer patients treated with neoadjuvant chemotherapy. Medicine.

[26] Wen W., Xuan D., Hu Y., Li X., Liu L., Xu D. (2019). Prognostic value of maximum standard uptake value, metabolic tumor volume, and total lesion glycolysis of positron emission tomography/computed tomography in patients with breast cancer: A systematic review and meta-analysis. PLoS ONE.

[27] Kitajima K., Miyoshi Y., Yamano T., Odawara S., Higuchi T., Yamakado K. (2018). Prognostic value of FDG-PET and DWI in breast cancer. Ann. Nucl. Med..

[28] Verma S., Chan J., Chew C., Schultz C. (2019). PET-SUV Max and Upstaging of Lung Cancer. Heart Lung Circ..

[29] Semrau S., Haderlein M., Schmidt D., Lell M., Wolf W., Waldfahrer F., Uder M., Iro H., Kuwert T., Fietkau R. (2015). Single-cycle induction chemotherapy followed by chemoradiotherapy or surgery in patients with head and neck cancer: What are the best predictors of remission and prognosis?. Cancer.

[30] Pan L., Gu P., Huang G., Xue H., Wu S. (2009). Prognostic significance of SUV on PET/CT in patients with esophageal cancer: A systematic review and meta-analysis. Eur. J. Gastroenterol. Hepatol..

[31] Li Y., Wu X., Huang Y., Bian D., Jiang L. (2020). ^18^F-FDG PET/CT in lung adenosquamous carcinoma and its correlation with clinicopathological features and prognosis. Ann. Nucl. Med..

[32] Lee E.J., Chang S.H., Lee T.Y., Yoon S.Y., Cheon Y.K., Shim C.S., So Y., Chung H.W. (2015). Prognostic value of FDG-PET/CT total lesion glycolysis for patients with resectable distal bile duct adenocarcinoma. Anticancer Res..

[33] Yoon H.J., Kim Y., Chung J., Kim B.S. (2019). Predicting neo-adjuvant chemotherapy response and progression-free survival of locally advanced breast cancer using textural features of intratumoral heterogeneity on F-18 FDG PET/CT and diffusion-weighted MR imaging. Breast J..

[34] Molina-García D., García-Vicente A.M., Pérez-Beteta J., Amo-Salas M., Martínez-González A., Tello-Galán M.J., Soriano-Castrejón Á., Pérez-García V.M. (2018). Intratumoral heterogeneity in ^18^F-FDG PET/CT by textural analysis in breast cancer as a predictive and prognostic subrogate. Ann. Nucl. Med..

[35] Huang S.Y., Franc B.L., Harnish R.J., Liu G., Mitra D., Copeland T.P., Arasu V.A., Kornak J., Jones E.F., Behr S.C. (2018). Exploration of PET and MRI radiomic features for decoding breast cancer phenotypes and prognosis. NPJ Breast Cancer.

